# SARS-CoV-2 Spike Protein Mutation at Cysteine-488 Impairs Its Golgi Localization and Intracellular S1/S2 Processing

**DOI:** 10.3390/ijms232415834

**Published:** 2022-12-13

**Authors:** Yuichiro Yamamoto, Tetsuya Inoue, Miyu Inoue, Mana Murae, Masayoshi Fukasawa, Mika K. Kaneko, Yukinari Kato, Kohji Noguchi

**Affiliations:** 1 Laboratory of Molecular Targeted Therapy, Faculty of Pharmaceutical Sciences, Tokyo University of Science, 2641 Yamazaki, Noda 278-8510, Chiba, Japan; 2 Department of Biochemistry and Cell Biology, National Institute of Infectious Diseases, 1-23-1 Toyama, Shinjuku-ku, Tokyo 162-8640, Japan; 3 Department of Antibody Drug Development, Tohoku University Graduate School of Medicine, 2-1 Seiryo-machi, Sendai 980-8575, Miyagi, Japan; 4 Department of Molecular Pharmacology, Tohoku University Graduate School of Medicine, 2-1 Seiryo-machi, Sendai 980-8575, Miyagi, Japan

**Keywords:** SARS-CoV-2, spike, cysteine, proteolysis, brefeldin A

## Abstract

The severe acute respiratory syndrome coronavirus 2 (SARS-CoV-2) spike protein binds to the cellular receptor—angiotensin-converting enzyme-2 (ACE2) as the first step in viral cell entry. SARS-CoV-2 spike protein expression in the ACE2-expressing cell surface induces cell–cell membrane fusion, thus forming syncytia. To exert its fusogenic activity, the spike protein is typically processed at a specific site (the S1/S2 site) by cellular proteases such as furin. The C488 residue, located at the spike–ACE2 interacting surface, is critical for the fusogenic and infectious roles of the SARS-CoV-2 spike protein. We have demonstrated that the C488 residue of the spike protein is involved in subcellular targeting and S1/S2 processing. C488 mutant spike localization to the Golgi apparatus and cell surface were impaired. Consequently, the S1/S2 processing of the spike protein, probed by anti-Ser-686-cleaved spike antibody, markedly decreased in C488 mutant spike proteins. Moreover, brefeldin-A-mediated endoplasmic-reticulum-to-Golgi traffic suppression also suppressed spike protein S1/S2 processing. As brefeldin A treatment and C488 mutation inhibited S1/S2 processing and syncytia formation, the C488 residue of spike protein is required for functional spike protein processing.

## 1. Introduction

Severe acute respiratory syndrome coronavirus 2 (SARS-CoV-2) structural proteins, such as spike, envelope, and membrane proteins, are transmembrane proteins synthesized in the perinuclear endoplasmic reticulum (ER), carried to the ER–Golgi intermediate compartment (ERGIC) between the ER and the Golgi apparatus, and mostly assembled into virions in the early secretory pathway, while some spike proteins are trafficked to the plasma membrane [1]. The spike protein is glycosylated in the ER and Golgi network, and some spike proteins are further cleaved at a multibasic site into S1 and S2 subunits by the Golgi enzyme or proprotease furin in the trans-Golgi network (TGN) [2]. The S1 domain contains a receptor-binding domain (RBD) that binds to the host cell-surface-expressed receptor protein, angiotensin-converting enzyme-2 (ACE2), while the S2 domain is responsible for viral entry and cell membrane fusion [3]. The second cleavage at the S2’ site in the SARS-CoV-2 spike, typically mediated by the cell-surface-expressed transmembrane protease TMPRSS2, followed the S1/S2 cleavage, influences viral infection and virus–cell membrane fusion. The S1/S2 cleavage causes a conformational change in the spike protein, and the second cleavage generates a fusion peptide at the S2’ site, which is critical for triggering membrane fusion [4]. Therefore, the SARS-CoV-2 spike protein has fusongenic activity. Since syncytia is formed in SARS-CoV-2-infected cells, intracellular traffic and spike expression in the cell surface are responsible for SARS-CoV-2 cytopathogenicity and coronavirus disease 2019 (COVID-19) pathogenesis [5,6].

The intracellular trafficking of transmembrane proteins synthesized in the ER through the secretory pathway is typically mediated by interactions between their cytoplasmic tails and the coat proteins COPI and COPII in the ER-to-Golgi apparatus [7]. COPI-mediated recycling within the Golgi apparatus causes interactions between the coronavirus spike and membrane proteins [8]. The SARS-CoV-2 spike protein has a dibasic KxHxx motif at the cytosolic tail, and mutating this motif reduces SARS-CoV-2 spike expression at the cell surface and syncytia formation; thus, it is critical for SARS-CoV-2 spike localization to the ERGIC/Golgi region, glycan processing, protease cleavage at S1/S2 site, and trafficking to the plasma membrane [1,9]. We have previously reported that mutating the C488 residue of the RBD abrogates the infectious role of the spike protein [10]. In this study, we demonstrated impaired plasma membrane and Golgi-apparatus-targeting C488 mutant spikes, which have intact cytoplasmic tails for intracellular trafficking. Consistently, the intracellular S1/S2 processing of C488 mutant spike proteins was suppressed. Overall, this study highlights the importance of C488 residue in the functional subcellular trafficking of spike proteins.

## 2. Results

### 2.1. C488 Is Important for the Fusogenic Activity and Cell-Surface Expression of the Spike Protein

**Figure 1:**
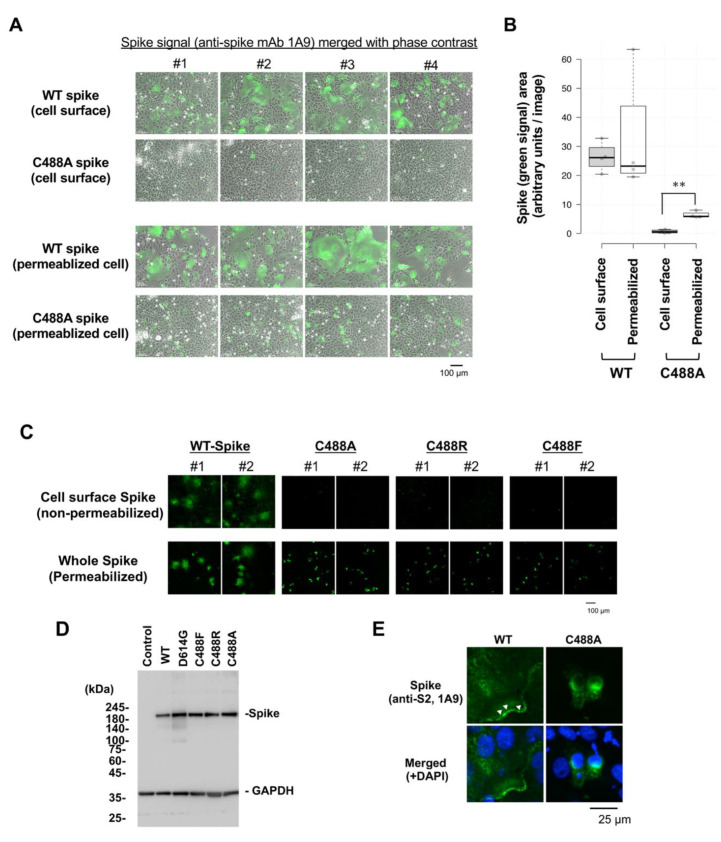
Loss of cell-surface expression of C488 mutant spike protein: (**A**) Wild-type (WT) and C488A mutant spike-expressing plasmids were transfected in Vero cells for 2 days. Cell surface spike protein was detected by anti-spike (1A9) antibodies and Alexa 488-conjugated secondary antibodies in non-permeabilized cells (**upper panels**). Intercellular spike protein was detected in Triton-X100 permeabilized cells (**lower panels**). (**B**) Green signals from spike-probed antibody in (**A**) were measured by ImageJ 1.44o. At least five images were analyzed in each setting. The spike-expressing area in C488A-expressing permeabilized cells was significantly larger than that in non-permeabilized cells. The Student’s *t*-test was performed to assess statistical significance. ** indicates *p* < 0.05. (**C**) Cell-surface expression of different C488 mutant spike proteins. WT and C488 mutant spike proteins were expressed in Vero cells for 2 days. Cell surface (**upper panels**) and whole intercellular (**lower panels**) spike proteins were detected, as in (A). The numbers #1 and #2 indicate two independent images. (**D**) Mutant spike protein expression was confirmed by Western blot analysis. GAPDH is shown as a loading control. (**E**) Subcellular localization of WT and C488A-mutant spike proteins in permeabilized Vero cells were analyzed by confocal microscopy. The green and blue signals represent spike proteins and DAPI-stained nuclei, respectively. The white arrowheads show spike proteins detected at plasma membrane.

Vero cells were transfected with a spike-expressing plasmid to compare the cell fusion activity of C488A mutant and wild-type (WT) spike proteins. WT spike protein induced multinuclear syncytia formation in Vero cells, but the C488A mutant spike protein did not (Figure 1A,B). Moreover, cell-surface C488A mutant spike in non-permeabilized cells was barely detected, while WT spike protein was easily detected (Figure 1A, upper images). However, both WT and C488A mutant spike proteins were clearly detected in Triton-X100-permeabilized Vero cells (Figure 1A, lower images). These observations indicate that most C488A mutant spike proteins were not localized to the plasma membrane.

To confirm the impaired plasma membrane localization of the spike protein, C488R and C488F mutant spike proteins were examined on the Vero cell surface. Cell-surface C488 mutant spikes were not observed (Figure 1C, upper images), but whole-cellular WT and C488 mutant spike proteins were equally visualized in permeabilized cells (Figure 1C, lower images). Western blot analysis showed that the WT and C488 mutant spike protein-expression levels were equal (Figure 1D). Confocal microscopy detected WT (Figure 1E, white arrowheads), but not C488A mutant, spike protein in the plasma membrane. The cell-surface expression levels were confirmed by flow cytometric analysis (Supplemental Figure S1). Collectively, these data indicate that C488 mutation compromised the plasma membrane targeting by the spike protein.

### 2.2. C488 Is Involved in Golgi-Targeting by the Spike Protein

**Figure 2:**
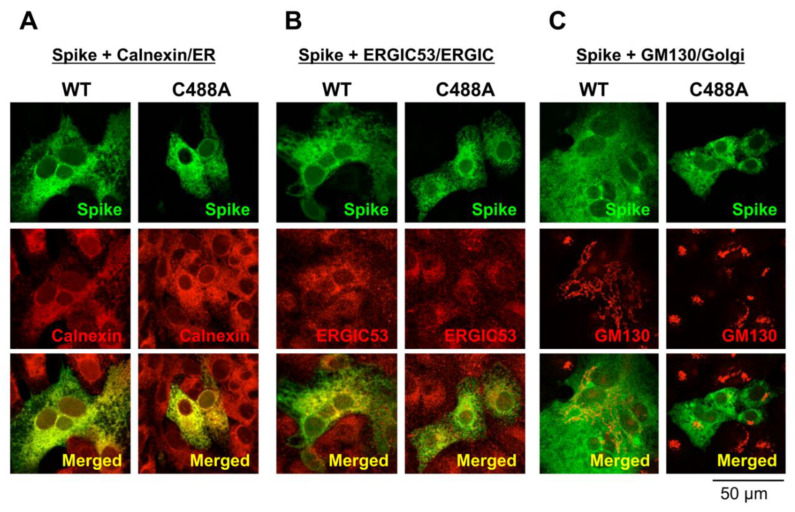
Subcellular localization of C488 mutant spike protein in Vero cells. Wild-type (WT) and C488A mutant spike-expressing plasmids were expressed in Vero cells for 2 days, and their subcellular co-localization with (**A**) calnexin, (**B**) ERGIC53, and (**C**) GM130 were analyzed by confocal microscopy. WT and C488A mutant spikes (green signals) were probed with anti-spike (1A9) antibodies and subcellular markers (red signals) used were: calnexin, ERGIC53, and GM130 as ER, ERGIC, and Golgi markers, respectively. A co-localized yellow signal is visualized in the merged images (lower panels).

Intracellular spike localization in Vero cells was examined by confocal microscopy. Green signals are spike proteins probed with the anti-spike monoclonal antibody 1A9. The co-localization at the ER and ERGIC were visualized by co-staining with calnexin and ERGIC 53, respectively (red signal; Figure 2A,B, respectively). Merged yellow signals suggest that both the WT and C488A mutant spike proteins were similarly detected in the ER and ERGIC. The co-localization at the Golgi apparatus was observed by co-staining with GM130 (Figure 2C). As described previously [11], WT spike expression induced Golgi fragmentation (Figure 2C, left panels); however, the C488A mutant spike was not co-localized with GM130 and did not induce Golgi fragmentation (Figure 2C, right panels). These data suggest that C488A mutation suppressed spike protein trafficking to the Golgi apparatus.

### 2.3. C488 Mutation Suppresses S1/S2 Processing of the Spike Protein

**Figure 3:**
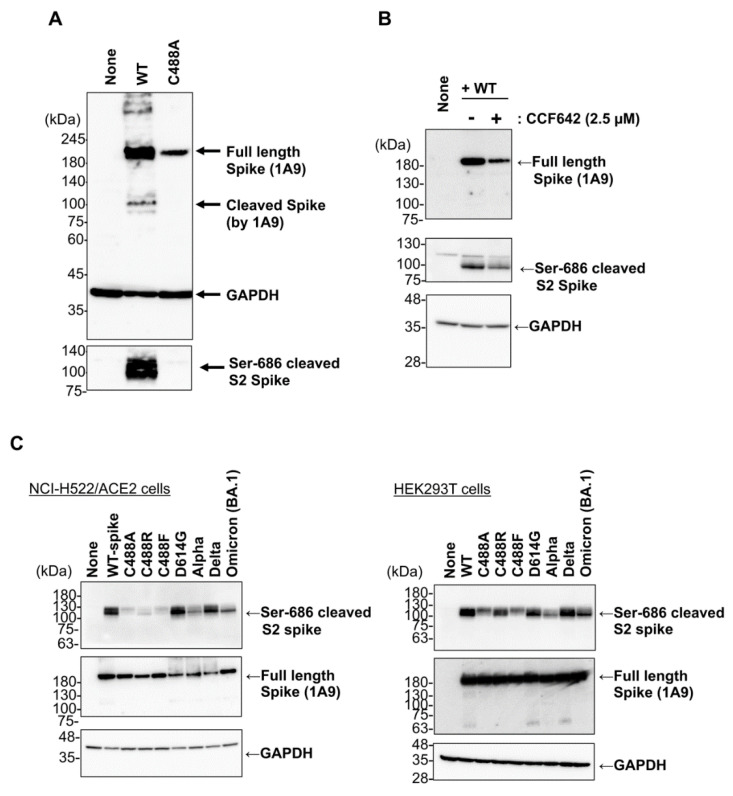
Defect of S1/S2 processing in C488 mutant spike proteins. (**A**) Spike-expressing plasmid was transfected into VeroE6/TMPRSS2 cells, and the cell lysate was prepared 20 h post-transfection. Full-length and Ser-686 cleaved spike proteins were probed with anti-spike (1A9) and cleaved SARS-CoV-2 spike (Ser686) antibodies, respectively. GAPDH is shown as a loading control. (**B**) Wild-type spike-expressing plasmid was transfected in HEK293 cells, which were treated with CCF642 for 13 h at 5 h post-transfection, followed by cell lysate preparation. Full-length and Ser-686 cleaved spike proteins were detected with anti-spike (1A9) and cleaved SARS-CoV-2 spike (Ser686) antibodies, respectively. (**C**) Each mutant spike-expressing plasmid was transfected in NCI-H522/hACE2 (**left panels**) and HEK293T (**right panels**) cells. After 20 h, cell lysates were prepared, and full-length and Ser-686 cleaved spike proteins were detected with anti-spike (1A9) and cleaved SARS-CoV-2 spike (Ser686) antibodies, respectively.

The SARS-CoV-2 spike protein can be processed by the proprotein convertase furin at the S1/S2 site. Furin is ubiquitously expressed in the Golgi apparatus and trans-Golgi network [12,13]. As the cell-surface expression of the C488A mutant was disturbed, we next compared S1/S2 processing of WT and C488A mutant spike proteins. The S1/S2 cleavage of the WT, but not the C488A mutant, spike protein was detected by an anti-Ser-686 cleaved spike monoclonal antibody (Figure 3A, bottom panel). As the C488A mutation abrogated disulfide-bond formation between C480 and C488, we tested the effect of an inhibitor of protein disulfide isomerases (PDI), which is required for protein folding in the ER, on the spike protein S1/S2 processing. After treatment with the PDI inhibitor CCF642, the whole spike protein level was slightly decreased, and subsequently, Ser-686 cleaved spike protein level was reduced. (Figure 3B). Thus, non-specific inhibition of disulfide-bond formation in the spike protein was not a major cause of the C488A mutation-mediated S1/S2 processing defect. Interestingly, a recent study showed that other cysteine mutants, such as C301F and C379F, reduced the S2 fragment spike protein, although the reason was not discussed [14]. Overall, these data suggested that the C488A mutation significantly attenuates ER–Golgi trafficking and S1/S2 processing of the spike protein. The mutant-mediated S1/S2 processing defect was analyzed in other variant spike proteins using other cell lines (Figure 3C). When C488 mutant and variant spike proteins were expressed in HEK293T cells and ACE2-transfected human lung adenocarcinoma NCI-H522 clone cells (NCI-H522/ACE2), Ser-686 cleavage in the C488A, C488R, and C488F spikes were reduced in both cell lines. The C488R and C488F mutations are naturally recorded in the SARS-CoV-2 database COV-GLUE (https://cov-glue.cvr.gla.ac.uk/index.php, accessed on 30 September 2022) with frequencies of 0.000002 and 0.000003, respectively. The Ser-686 cleavage in Wuhan-Hu-1, D614G, and Delta spike proteins were comparable, while those in Alpha and Omicron BA.1 were relatively low. Functional syncytium-inducing activity of Alpha variant spikes was observed in NCI-H522/ACE2 cells (Supplemental Figure S2); therefore, S1/S2 processing in cells may have a low impact on Alpha spike activity. The relatively low syncytium-inducing activity of Omicron-type spikes is consistent with the results of a recent study [15]. These results suggest that C488 mutation causes unique S1/S2 processing defects in the spike protein.

### 2.4. SARS-CoV-2 Membrane Protein Does Not Compensate C488 Mutation of Spike Protein

**Figure 4:**
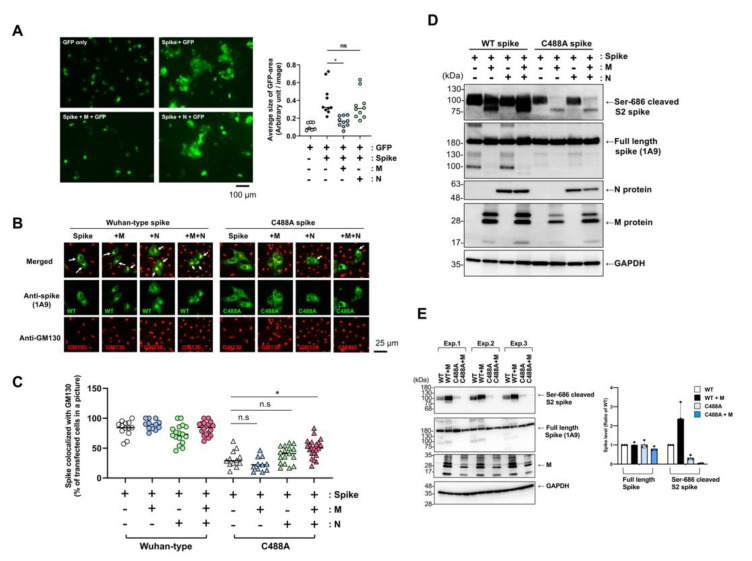
M protein did not affect C488A-mutant spike. (**A**) VeroE6/TMPRSS2 cells were co-transfected with spike-, M-, N-expressing plasmids with pEGFP-C1. The cells were fixed at 20 h post-transfection, and the green fluorescence protein (GFP) signals were photographed with conventional fluorescence microscopy. Areas of GFP-positive green cells were measured in each picture by ImageJ software, and average area size of GFP-positive cells in a picture was plotted in the right graph. One-way ANOVA with Tukey-HSD was performed to assess statistical significance. n.s. means “not significant”, and * indicates *p* < 0.01. (**B**) Spike-expressing plasmids were co-transfected with SARS-CoV-2 M or N protein-expressing plasmid in Vero cells, which were fixed and permeabilized. Co-localization of spikes (green) with GM130 (red) were analyzed by confocal microscopy. White arrows indicate complete co-localization (yellow) of spikes with GM130. (**C**) Co-localization of spikes and GM130 was examined in the transfected cells (*n* ≥ 156), and the ratio of co-localization is shown. One-way ANOVA with Tukey-HSD was performed to assess statistical significance. n.s. means “not significant”, and * indicates *p* < 0.01. (**D**,**E**) SARS-CoV-2 spike-expressing plasmid was co-transfected with M- or N-expressing plasmid in HEK293T cells. The cell lysate was prepared after 20 h, and full-length and Ser-686 cleaved spike proteins were detected by Western blotting with anti-spike (1A9) and cleaved SARS-CoV-2 spike (Ser686) antibodies, respectively. Triplicated transfected samples were analyzed (Exp. 1–3), and signal intensity was quantified by ImageJ (right graph in **E**).

Previous reports have shown that co-expression of the SARS-CoV-2 membrane (M) protein induces spike and nucleocapsid (N) protein accumulation in the Golgi apparatus [16,17]. WT spike-induced syncytium formation in VeroE6/TMPRSS2 cells was apparently suppressed by co-expression of M, but not by N, which confirmed functional interplay between the spike and M protein (Figure 4A). Therefore, we examined the effect of M protein co-expression on C488A localization in the Golgi apparatus. Immunofluorescence microscopic analysis showed that green signals of WT spike protein in most cells were co-detected with GM130 red signals, and the merged yellow signals were clear, showing Golgi-targeting of WT spike proteins in Vero cells 20 h post-transfection (Figure 4B (left images),C (quantitative results)). In contrast, co-localization of the C488A mutant spike protein with GM130 did not increase, even in the presence of M protein co-expression. Precise analysis indicated that GM130 red signals co-localized with the WT, but not the C488A mutant, spike signals (Supplemental Figure S3). However, co-expression of N and M combination slightly, but statistically significantly, enhanced co-localization of C488A mutant spike with GM130 (Figure 4B (right images),C (quantitative results)). Since Golgi localization affects S1/S2 processing, we examined the effect of M and N protein co-expression on spike protein S1/S2 processing (Figure 4D). The co-expression of M protein decreased S1/S2 processing (cleavage at Ser-686) of the C488A mutant spike, and co-expression of N protein appeared to show little impact on S1/S2 processing (Figure 4D). Quantitative, triplicated experiments indicated that M protein might increase S1/S2 processing of WT spike protein, but conversely decrease that of C488A mutant spike protein (Figure 4E). Full-length spike protein levels were comparable in the presence of M protein. Although previous studies revealed a critical role of the C-terminal cytoplasmic tail of the spike protein in ER–Golgi retention [9,16], our data suggest that the C488 mutation may affect spike protein targeting to the Golgi apparatus and functional interplay with M protein.

### 2.5. Brefeldin A Inhibits SARS-CoV-2 Spike Protein Processing

**Figure 5:**
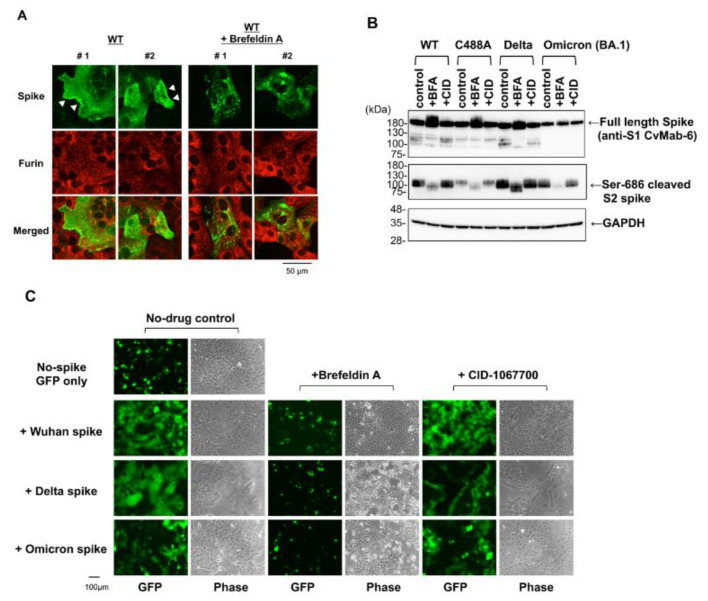
Brefeldin A (BFA) interferes with SARS-CoV-2 spike protein function. (**A**) Vero cells were transfected with spike-expressing plasmid. The cells were treated with or without 1 µM BFA for an additional 15 h at 9 h post-transfection. The spike protein localization in permeabilized cells was examined using confocal microscopy. The numbers #1 and #2 indicate two independent images. White arrowheads indicate plasma membrane localization of spike protein. (**B**) HEK293T cells were transfected with spike-expressing plasmid. The cells were treated with 1 µM BFA and 1 µM CID-1067700 (CID) for additional 19 h at 9 h post-transfection. Full-length and Ser-686 cleaved spike proteins were detected by Western blotting with anti-spike (1A9) and cleaved SARS-CoV-2 spike (Ser686) antibodies, respectively. (**C**) VeroE6/TMPRSS2 cells were transfected with spike-expressing plasmid with pEGFP-C1. The cells were treated with BFA or CID-1067700 for an additional 15 h at 5 h post-transfection. The green fluorescence protein (GFP) signals and phase contrast cell morphologies were photographed with conventional fluorescence microscopy.

Brefeldin A (BFA) is a fungal metabolite that disrupts intracellular membrane traffic at the ER–Golgi complex junction [18]. Hackstadt et al. reported that BFA-mediated Golgi apparatus disruption inhibited viral replication [11]. To confirm the involvement of ER–Golgi trafficking in S1/S2 processing of spike proteins, we tested the effect of BFA and observed that BFA treatment disrupted WT spike plasma membrane localization (Figure 5A), but not furin localization (red signal). Further, Western blot analysis using anti-Ser-686 antibodies indicated that BFA treatment suppressed S1/S2 processing of every spike protein variant (Figure 5B). BFA treatment also affected the electrophoretic mobility of Ser-686 cleaved S2 protein on Western blot analysis, suggesting that BFA-mediated Golgi-targeting inhibition might suppress glycosylation maturation of the spike protein. The function of the spike protein after BFA treatment was examined using a cell–cell fusion assay. BFA treatment inhibited WT, Delta, and Omicron spike-mediated syncytium formation in VeroE6/TMPRSS2 cells (Figure 5C). The spike-mediated syncytium was not inhibited by CID-1067700, a Rab7 GTPase competitive inhibitor that blocks lysosomal trafficking during beta-coronavirus egress [19]. Thus, ER-to-Golgi trafficking and S1/S2 processing of spike proteins are critical for the functional targeting of spike proteins to the plasma membrane.

## 3. Discussion

SARS-CoV-2 infection causes cell–cell fusion to form cytopathic syncytia, which requires cell-surface expression of the viral spike protein and its binding to ACE2 expressed on the neighboring cell surface [6]. The disulfide bond between C480 and C488 in the spike protein stabilizes the conformation of the extended loop structure at the RBD, which is required for ACE2-binding [20,21]. Previously, we have reported SARS-CoV-2 spike protein function loss by mutation at C488 residue [10] and demonstrated the novel effects of C488 residue on spike protein subcellular targeting and its S1/S2 processing. As BFA inhibited both ER-to-Golgi traffic and the S1/S2 processing of spike protein, our data indicate that the C488 residue of spike protein is required for the functional processing of spike proteins in cells.

The SARS-CoV-2 spike protein is a type I transmembrane protein initially translocated into the ER, glycosylated in the ER and Golgi apparatus, and further processed into S1/S2 subunits by furin in the trans-Golgi network [2]. The spike protein’s C-terminal cytoplasmic tail, rather than the RBD, regulates intracellular trafficking and S1/S2 processing of the spike protein [1,9]. The non-canonical COPI-binding motif in the SARS-CoV-2 spike C-terminal tail is speculated as allowing more spikes to reach the surface [8], and the sub-optimal COPI-binding site of SARS-CoV-2 spike protein C-terminal tail appears to contribute to its efficient plasma membrane localization for syncytia formation [1,9]. The M protein, another SARS-CoV-2 viral protein, induces spike protein retention in the ERGIC and Golgi apparatus [1,16]. Another viral structural, protein N, is known to be responsible for COVID-19 virulence as it affects the rate of replication of the virus [22]. Interaction among the coronaviral structural proteins (N, E, S, and M), as well as a host membrane envelope obtained from the site of budding, is required for viral assembly [23]. A recent study has suggested a predominant role for M protein in controlling the spatial organization of the transmembrane proteins and initiating the assembly of SARS-CoV-2, and that the M protein is responsible for the recruitment of the N protein to the Golgi/ERGIC membranes [17]. Our data consistently showed that the M + N combination enhanced C488A mutant spike targeting to the Golgi, suggesting a possible role of interaction between M and N proteins in the spike protein traffic mechanism. M-protein-mediated spike protein retention at the ERGIC and Golgi is also dependent on the intact spike protein C-terminal cytoplasmic tail, and M protein cannot reverse C488 mutation-mediated ER-to-Golgi traffic defects. In contrast, M protein decreased C488A mutant spike S1/S2 processing although the precise mechanism is unknown. As the C-terminal tail, which is required for intracellular trafficking to both the Golgi and plasma membranes, is intact in the C488 mutant spike protein, C488 mutation might affect mechanisms other than the C-terminal-tail-involved ER–Golgi–plasma membrane traffic. Tien et al. showed that LYQD mutations at 611–614 residues alter intracellular spike protein trafficking and reduce cell-surface expression levels [24]. While the way in which the LYQD motif might coordinate spike protein maturation with ER export and ER retrieval signals in the C-terminus is unclear, these observations raise the possibility that mechanisms other than the spike C-terminal tail may be involved in the intracellular SARS-CoV-2 spike protein maturation process.

S1/S2 processing in Alpha- and Omicron (BA.1)-type spike proteins detected by Ser-686 cleaved spike antibodies was relatively decreased (Figure 3C). Recent studies suggest that P681H mutation acquisition may inhibit cleavage by furin at low pH levels [25]. Therefore, the decrease in Ser-686 cleaved S2 spike protein levels in Alpha and BA.1 spike proteins may be due to the 681H mutation although the C488 mutants in this study do not have the 681H mutation. Consistent with this, the RBD is a determinant of reduced S1/S2 proteolysis in the Omicron BA.1 spike [26], and the N-terminal domain of the Delta spike allosterically modulates S1/S2 processing [27]. Furthermore, similar to the C488 mutations in RBD, which disrupt the C480–C488 disulfide bond of the spike protein, Yamaoka et al. recently showed that disulfide-bond formation is critical for RBD functioning, and C488A and C379A mutations lost their ACE2-binding ability [28]. In addition, Zhang et al. showed that the C301F and C379F mutations eliminate the C291–C301 and C379–C432 disulfide bonds located in the S1 N-terminal domain and RBD, respectively, thus causing the S1/S2 processing loss of the spike protein [14]. As S1/S2 processing was still detected during CCF642 treatment, a PDI inhibitor that blocks disulfide-bond-mediated proper protein folding in the ER (Figure 3B), the non-specific inhibition of disulfide bond formation would not be a major cause of the S1/S2 processing loss. These findings, along with our data here, might imply an important role for the RBD in the S1/S2 processing of spike protein in cells, and further studies will be needed to elucidate this mechanism.

## 4. Materials and Methods

### 4.1. Cells, Plasmids, and Reagents

HEK293T, Vero (ATCC, Cat#CCL-81), and VeroE6/TMPRSS2 (JCRB, Cat#JCRB1819) cells were cultured in Dulbecco’s Modified Eagle’s Medium (DMEM) supplemented with 7.5% (*v*/*v*) fetal bovine serum (FBS) and 25 µg/mL kanamycin. NCI-H522 (ATCC, Cat#CRL-5810) cells were cultured in RPMI1640 medium supplemented with 7.5% (*v*/*v*) FBS and 25 µg/mL kanamycin. NCI-H522/ACE2 cells were generated by isolating human ACE2-DYK-expressing plasmid (synthesized by GenScript Japan Inc., Tokyo, Japan)-transfected stable cells. The plasmids used are listed in Table 1. BFA was purchased from Fujifilm Wako Pure Chemical Corp. (Osaka, Japan). CCF642 (Cat#S8281) and CID-1067700 (Cat#E0135) were purchased from Selleck Biotech Corp. (Tokyo, Japan).

### 4.2. Transfection

Synthetic cDNA with human codon optimization to express SARS-CoV-2 spike and membrane proteins were purchased from GenScript and cloned into the expression plasmid pcDNA3.1. Plasmid DNA was transfected using PEIpro^®^ transfection reagent (Cat#101000017, Polyplus Transfection, New York, NY, USA) for Western blot and immunofluorescence analyses, as described previously [10].

### 4.3. Immunofluorescence Microscopic Analysis and Antibodies

Immunofluorescence analysis was performed as described previously [10]. Fluorescence-conjugated secondary antibodies (goat anti-mouse IgG (H + L) cross-adsorbed with Alexa Fluor^®^ 488, Cat#A-11001, and donkey anti-rabbit IgG (H + L) cross-adsorbed with Alexa Fluor^®^ Plus 594, Cat#A-32754) were purchased from Thermo Fisher Scientific (Life Technologies Corp.; Carlsbad, CA, USA), and antifade (Vectashield Vibrance Antifade Mounting Medium with DAPI, Cat#H-1700) was purchased from Vector Laboratories Inc. (Burlingame, CA, USA). Conventional immunofluorescence images were captured using a BZ-9000 microscope (KEYENCE; Osaka, Japan). The green fluorescence protein (GFP) signal was measured using ImageJ software (Rasband, W.S., ImageJ, U.S. National Institutes of Health; Bethesda, MD, USA, http://imagej.nih.gov/ij/, 1997–2012, accessed on 14 September 2022). Confocal microscopy was performed using a TCS SP8 confocal laser scanning microscope (Leica Microsystems GmbH; Wetzlar, Germany) and Leica Application Suite X software (Leica). TIFF images were merged using Adobe Photoshop CS4 Extended software (Adobe Systems Inc., San Jose, CA, USA). Anti-calnexin (Cat#PM060MS) and anti-GM130 (Cat#PM061MS) antibodies were purchased from Medical & Biological Laboratories Co., Ltd. (Aichi, Japan). Anti-ERGIC53/LMAN1 (Cat#EPR6979) and anti-furin (Cat#EPR14674) antibodies were purchased from Abcam K.K. (Tokyo, Japan).

### 4.4. Hybridoma Production of mAbs against SARS-CoV-2

Two 6-week-old female BALB/c mice were purchased from CLEA Japan (Tokyo, Japan) and housed under specific pathogen-free conditions. The Animal Care and Use Committee of Tohoku University approved all of the animal experiments (permit number: 2019NiA-001). Each BALB/c mouse was intraperitoneally (i.p.) immunized with 100 μg N-terminal His-tagged S1 spike protein of SARS-CoV-2 (Ray Biotech, Cat#230-01102) using Imject Alum (Thermo Fisher Scientific Inc., Walthan, MA, USA). The procedure included three additional immunization procedures (100 μg/mouse), followed by a final booster intraperitoneal injection (100 μg/mouse) 2 days before harvesting the spleen cells, which were subsequently fused with P3U1 cells (ATCC, CRL-1597) using polyethylene glycol 1500 (PEG1500; Roche Diagnostics; Indianapolis, IN, USA). The hybridomas were then grown in RPMI medium supplemented with hypoxanthine, aminopterin, and thymidine for selection (Thermo Fisher Scientific, Inc.). The culture supernatants were screened using an enzyme-linked immunosorbent assay (ELISA) for detecting SARS-CoV-2 S1. Clone CvMab-6 culture supernatant using hybridoma-SFM medium (Thermo Fisher Scientific Inc.) was purified using Ab-Capcher (ProteNova, Kagawa, Japan).

### 4.5. Immunoblotting

Cells were lysed using radioimmunoprecipitation assay (RIPA) buffer (Cat#16488-34, Nacalai tesque, Inc., Kyoto, Japan)-containing protease inhibitors. Proteins were resolved by electrophoresing on a 5–20% gradient sodium dodecyl sulfate polyacrylamide gel and electrotransferred to an Immobilon-P membrane (Cat#IPVH00010, EMD Millipore; Billerica, MA, USA). The primary antibodies against SARS-CoV-2 anti-spike (Cat#GTX632604, clone 1A9), anti-nucleocapsid (Cat#GTX635679, HL344), and anti-membrane (Cat#GTX636245, clone HL1087) proteins were purchased from GeneTex Inc. (Irvine, CA, USA). Cleaved SARS-CoV-2 spike protein (Ser686) antibodies (Cat#84534) and anti-mouse IgG–HRP (Cat#7076) were purchased from Cell Signaling Technology, Inc. (Danvers, MA, USA). Anti-rabbit IgG–HRP (Cat#NA934) was purchased from Cytiva™ (Tokyo, Japan). Anti-glyceraldehyde 3-phosphate dehydrogenase (anti-GAPDH; Cat#171-3, clone 3H12) was purchased from Medical & Biological Laboratories Co., Ltd. (Aichi, Japan). Immunoblot signals were developed using EzWestLumi plus^®^ (Cat#WES-7120, ATTO Corp.; Tokyo, Japan) and recorded with an ImageQuant LAS4000 mini-image analyzer (GE Healthcare Japan Corp.; Tokyo, Japan).

### 4.6. Statistical Analysis

Quantitative results are presented as mean ± standard deviation (SD) from a minimum of three samples (*n* ≥ 3). The Student’s *t*-test or one-way ANOVA was used to evaluate the significance of differences between the experimental and control groups with similar variances. Differences were considered statistically significant at *p* < 0.05. Each experiment was independently repeated at least twice, and similar results were obtained. The statistical analysis and graphing were performed using GraphPad Prism 9 software.

## Figures and Tables

**Table 1:** Plasmids used in this study.

Plasmid Names	Remarks, Protein Sequence and Mutation
pEGFP-C1	BD Biotech Clontech
pcDNA3.1–spike/WT	Wuhan Hu-1 spike, YP_009724390.1
pcDNA3.1–M	Wuhan Hu M, YP_009724393
pcDNA3.1 SARS-CoV-2 N	pcDNA3.1 SARS-CoV-2 N was a gift from Jeremy Luban (Addgene plasmid # 158079; http://n2t.net/addgene:158079, accessed on 15 November 2022; RRID:Addgene_158079) [29]
pcDNA3.1–spike/D614G	D614G
pcDNA3.1–spike/C488A	C488A
pcDNA3.1–spike/C488R	C488R
pcDNA3.1-spike/C488F	C488F
pcDNA3.1–spike/Alpha	H69/V70-del, Y144-del, N501Y, A570D, D614G, P681H, T716I, S982A, D1118H
pcDNA3.1–spike/Delta	L5F, T19R, E156G, F157/R158-del, L452R, T478K, D614G, P681R, D950N
pcDNA3.1–spike/Omicron (BA.1)	A67V, H69/V70-del, T95I, G142D, del143–145, ins214EPE, NL211–212I, G339D, S371L, S373P, S375F, K417N, N440K, G446S, S477N, T478K, E484A, Q493R, G496S, Q498R, N501Y, Y505H, T547K, D614G, H655Y, N679K, P681H, N764K, D796Y, N856K, Q954H, N969K, L981F
pcDNA3.1-hACE2-DYK	GenScript, Cat No:OHu20260D

## Data Availability

Not applicable.

## Supplementary Materials

The following supporting information can be downloaded at: https://www.mdpi.com/article/10.3390/ijms232415834/s1.
