# Supplementary material for: SARS-CoV-2 Spike Protein Mutation at Cysteine-488 Impairs Its Golgi Localization and Intracellular S1/S2 Processing
Source: Int J Mol Sci. 2022 Dec 13;23(24):15834. doi: 10.3390/ijms232415834 (PMC9779352; doi:10.3390/ijms232415834)
Supplement: Supplementary file 1 [file ijms-23-15834-s001.zip › Supplemental Figures.pdf]

Supplemental figure S1

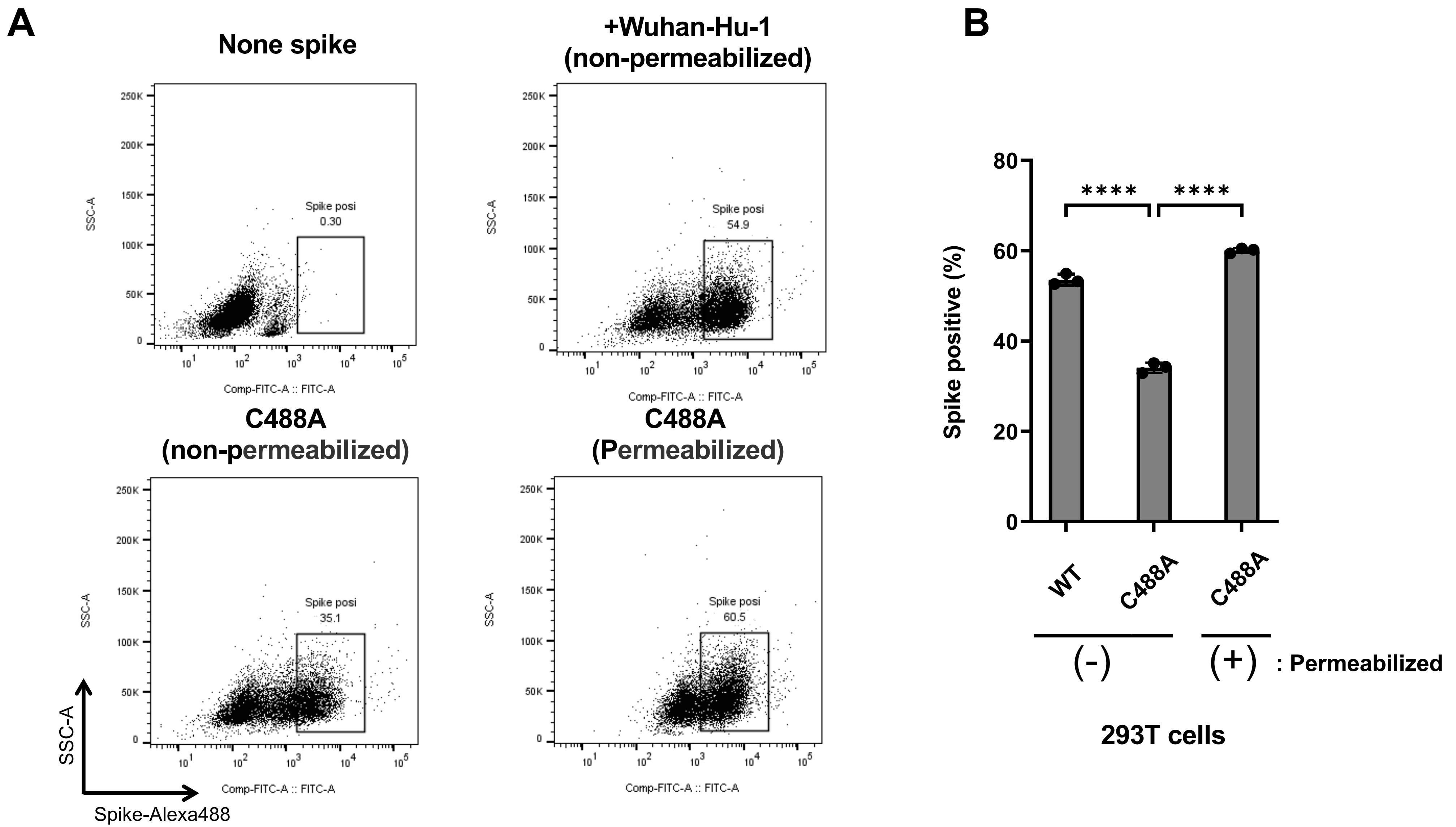

Supplemental figure S2

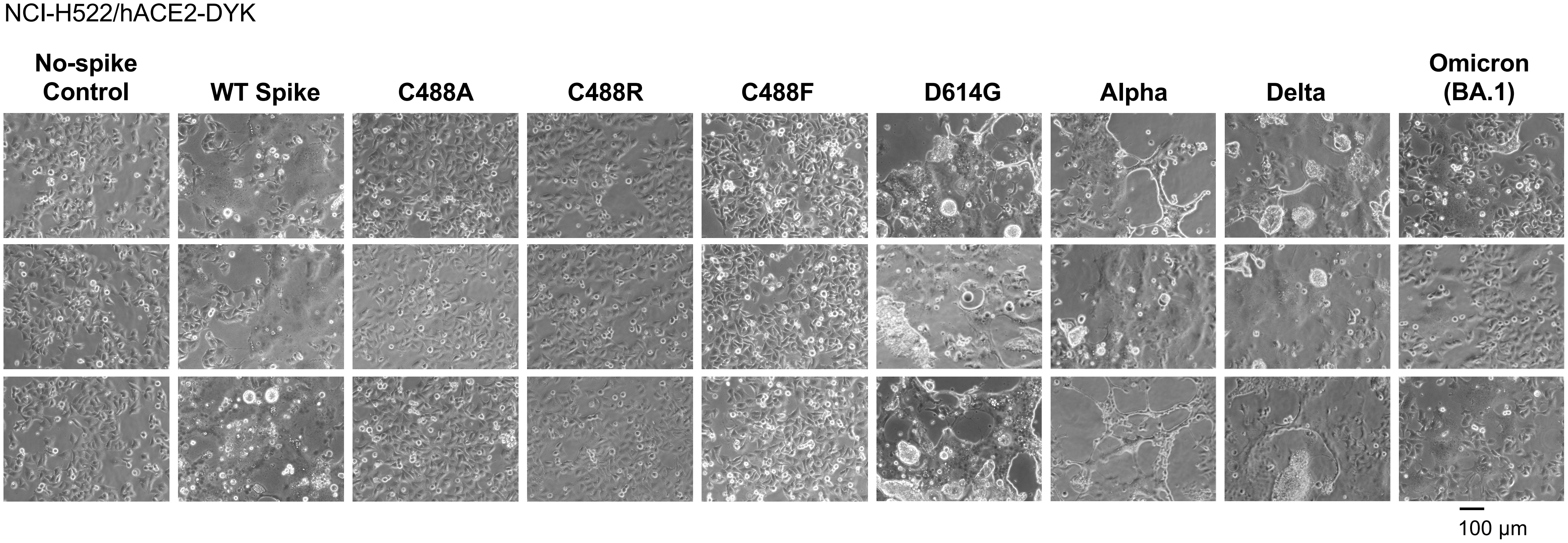

Supplemental figure S3

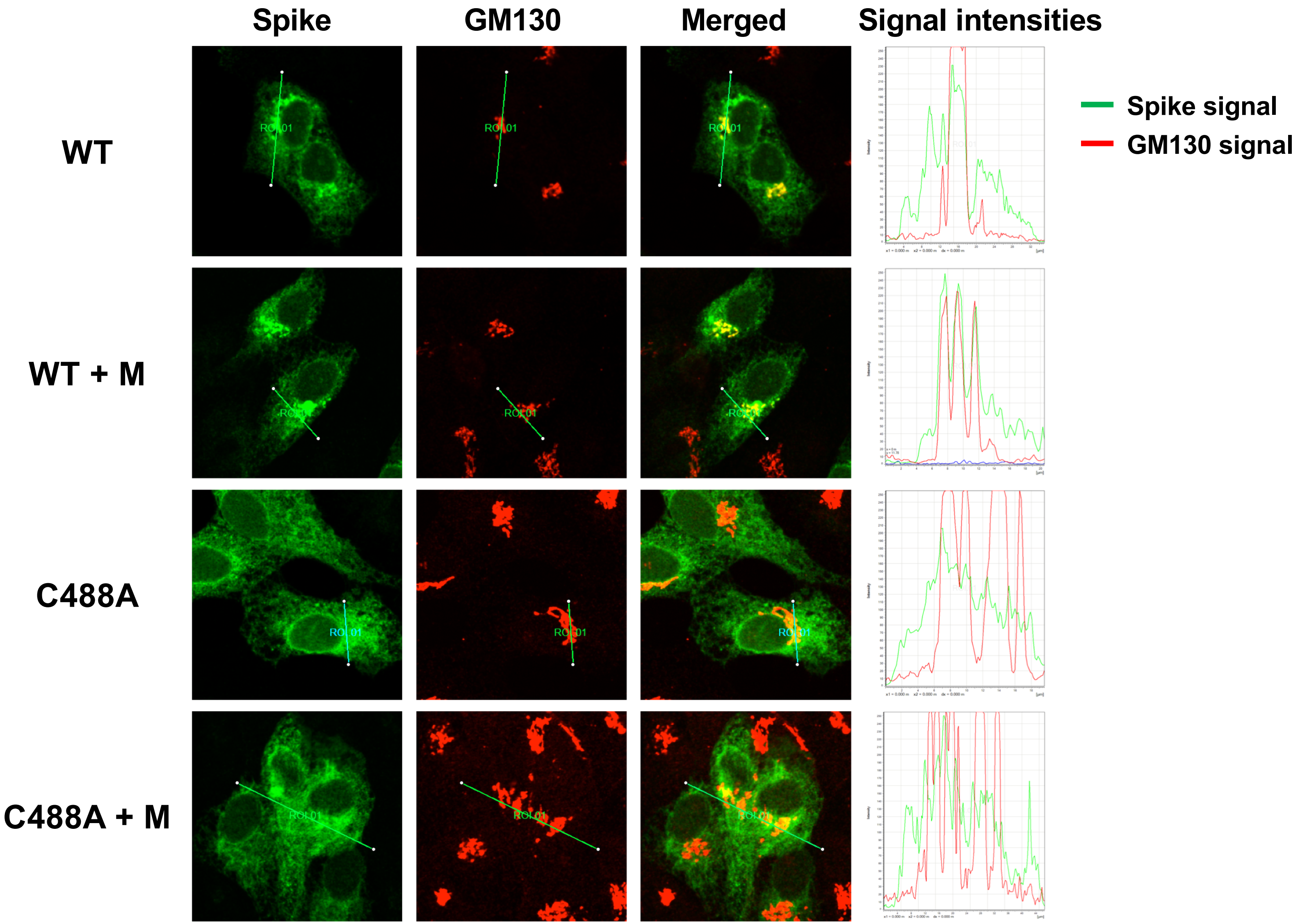

Figure legends for supplemental figures.

Figure S1: Detection of cell surface wild-type (Wuhan-Hu-1) and C488A mutant spike protein in 293T cells by flow cytometer. Non-permeabilized or Triton-X-100-permeabilized cells transfected either wild-type or C488A mutant spike-expressing plasmid were stained by anti-spike monoclonal antibody1A9. Anti-spike antibody-bound cells were probed with secondary anti-mouse IgG-Alexa488 conjugated. Cell surface-spike detected cells were gated (Figure S1A) and ratio of them are counted (Figure S1B). The ratio of C488A spike expressing cells was increased in permeabilized cells compared with that in non-permeabilized cells, suggesting that cell surface targeting of spike protein was compromised by C488A mutation.

Figure S2: Cellular syncytium induction of variant spike proteins was examined in NCI-H522/ACE2 cell lines. At 20h posttransfection, spike-induced syncytium was photographed. Three images of independent filed were shown. Syncytia was not induced in C488A, C488R and C488F spike-transfected cells. Severe syncytium and cytopathic effect were observed in D614G, Alpha and Delta spike-transfected cells.

Figure S3: Signal intensities in Figure 4A were measured along the lines and shown in the graph on the right.
